# Zinc Status and Autoimmunity: A Systematic Review and Meta-Analysis

**DOI:** 10.3390/nu10010068

**Published:** 2018-01-11

**Authors:** Alessandro Sanna, Davide Firinu, Patrizia Zavattari, Paolo Valera

**Affiliations:** 1 Department of Civil, Environmental Engineering and Architecture, University of Cagliari, 09123 Cagliari, Italy; alesanna@unica.it; 2 Department of Medical Sciences and Public Health, Monserrato Campus, University of Cagliari, 09042 Monserrato, Italy; davidefirinu@yahoo.it; 3 Unit of Biology and Genetics, Department of Biomedical Sciences, University of Cagliari, 09042 Monserrato, Italy

**Keywords:** zinc, autoimmunity, meta-analysis, multiple sclerosis, type 1 diabetes, rheumatoid arthritis

## Abstract

Zinc is an essential trace element for living organisms and their biological processes. Zinc plays a key role in more than 300 enzymes and it is involved in cell communication, proliferation, differentiation and survival. Zinc plays also a role in regulating the immune system with implications in pathologies where zinc deficiency and inflammation are observed. In order to examine the experimental evidence reported in the literature regarding zinc levels in the body of patients with autoimmune disorders compared to control individuals, a systematic review and meta-analysis were performed. From 26,095 articles identified by literature search, only 179 of them were considered potentially relevant for our study and then examined. Of the 179 articles, only 62 satisfied the inclusion criteria. Particularly for Fixed Model, Zn concentration in both serum (mean effect = −1.19; confidence interval: −1.26 to −1.11) and plasma (mean effect = −3.97; confidence interval: −4.08 to −3.87) samples of autoimmune disease patients was significantly lower than in controls. The data presented in our work, although very heterogeneous in the manner of collecting and investigating samples, have proved to be extremely consistent in witnessing a deficiency of zinc in serum and plasma of patients compared to controls.

## 1. Introduction

Zinc is an essential trace element for living organisms and their biological processes [1,2]. The body cannot accumulate zinc and it is, therefore, essential to take this element consistently in the diet. Although dietary zinc levels vary substantially, eukaryotic cells need to maintain intracellular zinc homeostasis to ensure its proper function. This homeostasis is regulated in mammals by import and export processes, vesicle retaining zinc (zincosomes) and association to metallothioneins (MTs) [3,4]. Zinc plays a key structural or catalytic role in more than 300 enzymes and is involved at all levels of cellular signal transduction. Zinc is involved in cell communication, cell proliferation, differentiation and survival. Therefore, zinc also plays a key role in regulating the immune system, both innate and adaptive, with consequent implications in pathologies where zinc deficiency and inflammation are observed.

The understanding of zinc’s importance in human health unfortunately begun only in the 1960s. Zinc deficiency is associated with a decline in the immune system, with inflammation leading to chronicity [5]. In addition, dietary zinc deficiency was considered to be very rare, although it affects 20–25% of the world’s population [6,7]. Data from the World Health Organization [8] report that zinc deficiency is the fifth largest health risk factor in developing countries and the eleventh in the world [9]. Rarely is zinc deficiency seen as a serious deficit; more frequently it is seen as a less accentuated deficit. Patients with severe deficits present: lymphopenia, decreased ratio between T helper (Th) to cytotoxic T cells, reduced natural killer (NK) cell activity, and increased monocytes cytotoxicity. This condition characterizes the malabsorption autosomal recessive syndrome, Acrodermatitis enteropathica, due to a mutation of a zinc-importing protein, ZIP4 [10].

Less accentuated zinc deficiency states can be caused by nutritional deficits due, for example, to a diet high in lignin and phytates, in vegetarians and vegans, chelating zinc, so preventing its proper absorption [11,12]. This state is characterized by slight weight loss, rough skin, oligospermia and hyperammoniaemia [13].

Several clinical trials of zinc supplementation have been conducted in patients with zinc deficit suffering from various pathologies (viral, bacterial and parasitic infections or autoimmune diseases) [14], or as vaccine supplements [15]. Although there is countless evidence supporting the fact that controlled zinc supplementation can prevent chronic inflammation and other zinc deficiency-related illnesses, or even improve symptoms (as seen in both humans and animal models), to date zinc supplementation does not fall into commonly used medical practices in risk subjects/populations. The purpose of this study was to examine the experimental evidence reported in the literature over the last 40 years regarding zinc levels in the body of patients with autoimmune disorders compared to control individuals. The biological matrices for which it was possible to collect enough bibliographic material to perform a meta-analysis were predominantly serum and plasma; to a lesser extent data were collected on urine, hair and spinal fluid.

## 2. Materials and Methods

### 2.1. Search Strategy

In order to select the included studies, a literature search was undertaken of PubMed, Cochrane Central Register of Controlled Trials, Web of Science and Science Direct databases from inception to 23 January 2017 and without any limitation of on year of publication. Keywords used were zinc and ((dietary or supplement) or (serum or plasma)) and (autoimmune disease or autoimmunity). Typing, in the search window, the keywords in the databases chosen, without any restrictions, the result was a list of publications for which only the title, authors and abstracts were available. After eliminating the duplicates the titles and abstracts of the remaining articles were read and those not relevant for the purpose of this meta-analysis were excluded. After this step, the entire manuscript of each remaining paper, defined as eligible, was read, thus excluding those that did not fall within the criteria defined in the “study eligibility criteria” (Section 2.3, below). Finally, the papers that satisfied all selection criteria have been included in the meta-analysis.

**Figure 1:**
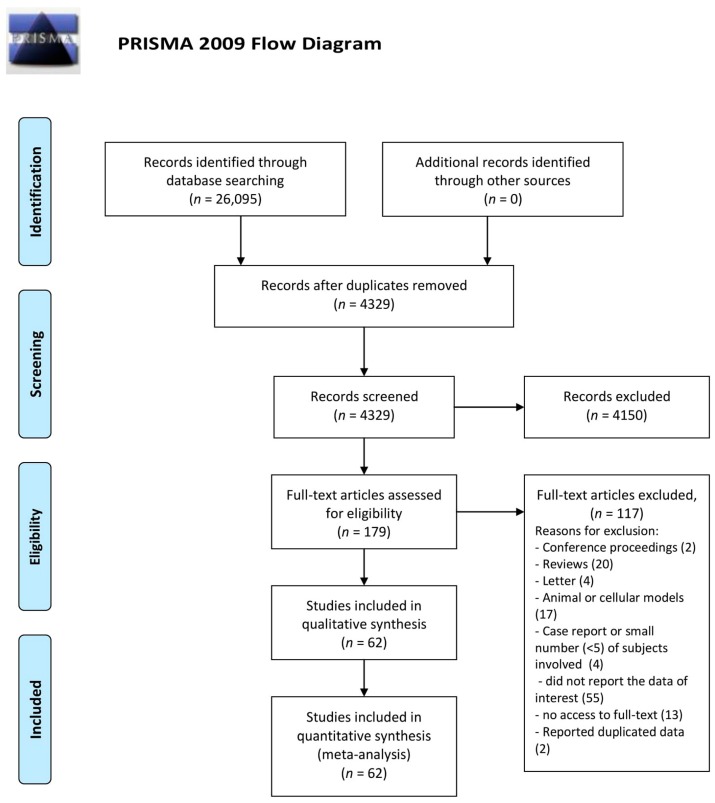
PRISMA flowchart diagram describing the systematic reviews process. PRISMA = Preferred Reporting Items for Systematic reviews and Meta-Analyses.

Full search details for all databases are presented in Table S1 (Supplemental Material). This study was performed according to Preferred Reporting Items for Systematic reviews and Meta-Analyses (PRISMA) requirements [16,17]. The standard flowchart, which describes the process selection, is reported in Figure 1.

### 2.2. Study Selection

All reference lists were downloaded for elimination of duplicates. Furthermore, title and abstract of each reference were screened by two independent reviewers to perform the eligibility assessment of full text for this review. Study eligibility was restricted to human studies and English language.

### 2.3. Study Eligibility Criteria

Observational studies, prospective and retrospective studies, case-control studies or randomized controlled trials (RCTs) investigating the relationship between zinc status and autoimmune diseases were eligible for inclusion. In particular, only diseases for which the autoimmunity was a franc condition were included in this study. The American Autoimmune Related Diseases Association (AARDA) [18] was consulted to verify that the diseases considered in this study were really autoimmune diseases.

The studies were selected if zinc concentration in biological samples or dietary/supplemental zinc were an index of zinc status.

Moreover, the presence of both number of subject involved (≥5) and the statistical parameters were taken into account for the meta-analysis process. On the other hand, letters, conference proceedings, reviews, duplicated data, data of both animal and cellular studies, and studies that did not indicate data of interest were excluded. Although studies on animals are not considered eligible for the meta-analysis process, a discussion on them was performed separately. Also, thirteen studies were excluded because we did not have access to the full text, maybe the most of these studies were published more than 20 years ago.

### 2.4. Study Quality Assessment and Data Extraction

The Newcastle-Ottawa Scale (NOS) for case-control studies was used to assess the quality of the included studies [19]. Ten full-text studies were excluded because control data was not complete in reference to both number of controls and their relationship with cases. Two independent reviewers extracted data from each eligible study. The data extracted included the type of study (observational studies, prospective and retrospective studies, case-control studies, randomized controlled trials), country, year of publication, sample size, age and sex of patients, autoimmune disease, zinc status in biological samples, type of samples, method of samples analysis, statistical method, standard deviation and statistical significance.

Table 1 reports the general characteristics of selected studies included in meta-analysis in reference to serum samples whereas Table 2 indicates the baseline characteristics for meta-analysis related to plasma samples. Furthermore, Table 3 reports the general information about the studies that are related to hair, urine and Cerebral Spinal Fluid (CSF) samples.

In most of the included studies, the measurement unit of the zinc concentration data were different for the same biological media. For this reason, all data of zinc in both serum and plasma samples were converted in μg/mL, whereas zinc data in hair samples were converted in μg/g.

### 2.5. Statistical Analysis

Data were processed by MetaEasy Excel add-in (Microsoft Corporation, Redmond, WA, USA), which used seven different methods. In particular, three methods refer to dichotomous data and four methods refer to continuous data. In order to improve their heterogeneity, data were grouped depending on different biological media: serum, plasma, hair, urine and cerebrospinal fluid. Means of zinc status in both patients and controls, standard deviations of zinc status in both patients and controls, number of subjects involved and *p*-value were used. *p*-value < 0.05 was considered as statistically significant.

Also, overall estimates of effect were performed with seven models: Fixed Effects (FE), DerSimonial-Laird (DL), Q method (Q), Maximum-Likelihood (ML), Profile—Likelihood (PL), *t*-Test (T) and Permutations method (PE). Heterogeneity was assessed by different measures: Cochrane’s Q, τ^2^ test, I^2^ test and H^2^M test. Publication bias was evaluated using funnel plot 3 considering the estimate of effects and their standard errors as a precision indicator (1/SE) [82].

## 3. Results

From 26,095 articles identified by literature search, 21,766 duplicates were deleted. After the screening of 4329 remaining articles, 4150 of them were excluded because they were irrelevant for our study. Afterwards, 179 papers were considered potentially relevant for our study but only 62 of them satisfied the inclusion criteria (see Section 2.4). Therefore, 117 articles were excluded and the reasons for their exclusion is shown in PRISMA diagram (Figure 1). In particular, 13 of the 117 papers were excluded because, with the means available to the Italian library system, it was not possible to retrieve them. However, the 13 papers are listed in Table S2 (Supplemental Material).

### 3.1. Zn Status and Autoimmune Diseases

The relationship between Zn status and autoimmune diseases has been investigated by many authors since the 1970s. The 62 studies included in the meta-analysis were case-control studies. Also, all of them were published between 1975 and 2017 and they are related to different autoimmune diseases. Indeed, 22 studies describe the zinc status in Type 1 Diabetes (T1D), 18 full-text are related to Rheumatoid Arthritis (RA), 7 articles investigated zinc status in Multiple Sclerosis (MS) patients while 15 papers are related to other diseases among which Alopecia Areata (AA), Systemic Lupus Erythematosus (SLE), Pemphigus Vulgaris (PV), Autoimmune Hepatitis (AH), Celiac Disease (CD), Hashimoto Thyroiditis (HT), Sjogren’s syndrome (SS), Juvenile Idiopathic Arthritis (JIA).

### 3.2. Zn Status in Serum Samples

The meta-analysis results show that, for all models, Zn concentration in serum of autoimmune disease patients was significantly lower than controls (FE: mean effect = −1.19 and confidence interval: −1.26 to −1.11; DL: mean effect = −1.29 and confidence interval: −1.91 to −0.67; Q: mean effect = −1.29 and confidence interval: −1.91 to −0.67; ML: mean effect = −1.29 and confidence interval: −1.96 to −0.63; PL: mean effect = −1.29 and confidence interval: −1.97 to −0.61; T: mean effect = −1.29 and confidence interval: −1.99 to −0.60; PE: mean effect = −1.29 and confidence interval: −2.95 to −0.49). Indeed, 70% of the articles considered show that patients have a zinc deficiency compared to the control group.

**Figure 2:**
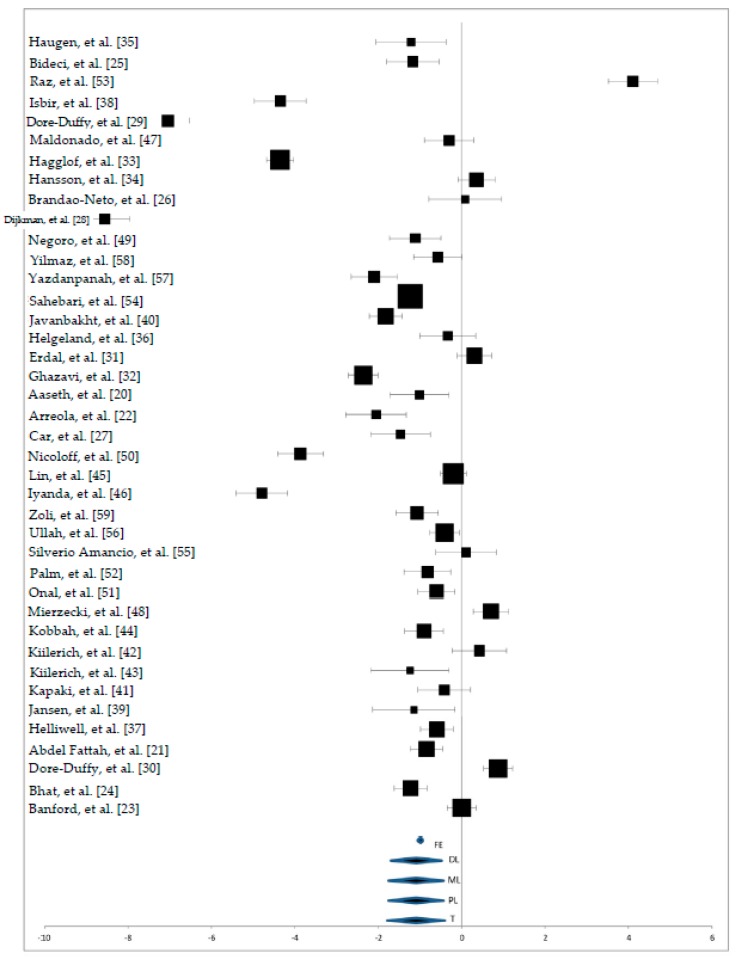
Forest plot of zinc status in serum samples. FE: Fixed Effects; DL: DerSimonial-Laird; ML: Maximum-Likelihood; PL: Profile—Likelihood; T: *t*-Test.

Regarding the heterogeneity of data, the elaboration shows the following results: Cochrane Q = 2589.53; τ^2^ = 3.88 (for DL model); τ^2^ = 4.52 (for ML and PL models); *I*^2^ = 98.49%; H^2^M = 65.39. Forest plot in Figure 2 shows the study effects for each study and the overall estimates effects. Despite high heterogeneity, overall estimates effects were positive for all models. Moreover, overall effects that were calculated with FE models were more efficient than effects calculated with the other models.

### 3.3. Zn Status in Plasma Samples

As already seen for serum, also in plasma samples the meta-analysis results show that, for all models, Zn concentration in the serum of autoimmune disease patients was significantly lower than controls. Indeed, for 62% of the articles included in the meta-analysis process, patients had lower zinc concentrations than controls.

**Figure 3:**
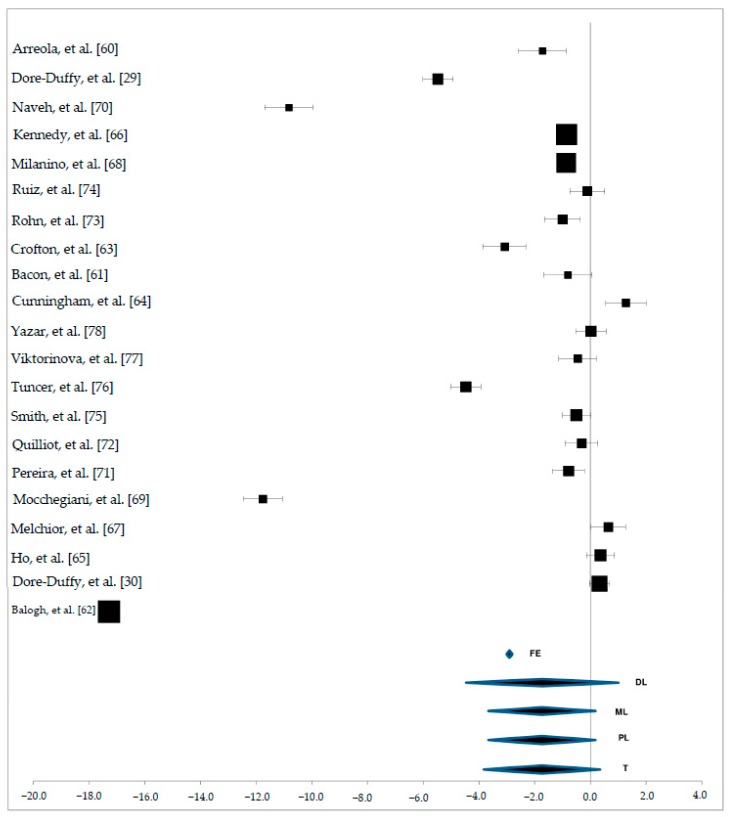
Forest plot of zinc status in plasma samples.

As shown in Figure 3, only FE model overall estimates effects could be considered favorable (FE: mean effect = −3.97 and confidence interval: −4.08 to −3.87). Even in this case, as in the previous one, data was highly heterogeneous.

### 3.4. Zn Status in Hair, Urine and Cerebrospinal Fluid Samples

In reference to Zn hair concentration, only FE model overall estimates effects could be considered favorable (FE: mean effect = −2.49 and confidence interval: −2.72 to −2.28). However, heterogeneity was considerable and the number of studies was limited. On the other hand, no significant variations in urinary and CSF zinc were observed between patients and controls.

### 3.5. Publication Bias

**Figure 4:**
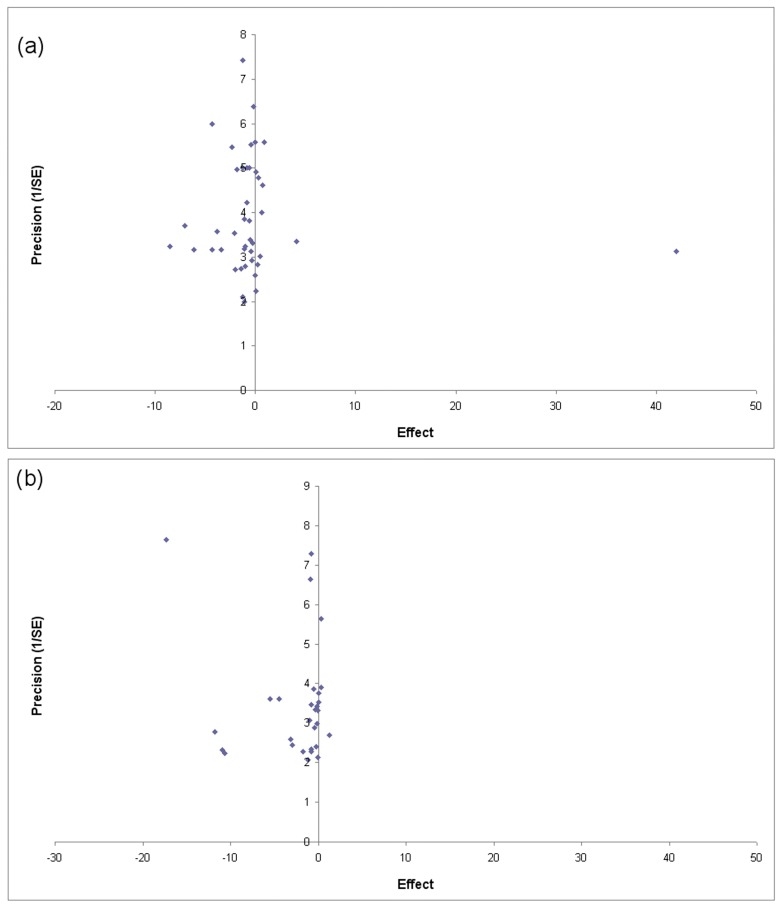
(**a**) Funnel plot for meta-analysis related to serum Zn; (**b**) Funnel plot for meta-analysis related to plasma Zn.

To evaluate the presence of publication bias, Funnel Plots were calculated. As shown in Figure 4, in both meta-analysis related to serum Zn and plasma Zn, it is possible to observe the presence of bias in the selected literature.

## 4. Discussion

This review of the literature on the possible linkage between zinc levels (especially in serum and plasma) and autoimmune diseases has revealed a huge amount of studies on this subject, although the selection due to meta-analysis methods has narrowed the final analysis to 62 publications, temporally distributed as shown in Figure S1 (Supplemental Material).

As expected, the data presented in this review, although very heterogeneous in the manner of collecting and investigating samples, etc., have proved to be extremely consistent in witnessing a deficiency of zinc in serum and plasma of patients compared to controls. A recurring question found in many studies was whether alterations in the homeostasis of this element represent the basis of the inflammatory status or consequences thereof.

As is well known, there are populations such as Finnish or Sardinian with polygenic predisposition to autoimmune diseases, in whom there has been a natural selection in favor of certain genetic loci, playing a role in the immune response. In particular, certain HLA (Humane Leucocyte Antigens) haplotypes, such as HLA-DR3-B18 in Sardinia and HLA-DR4 in Finland, are particularly frequent in those populations, terribly increasing the relative risk of developing multiple sclerosis [83], type 1 diabetes [84] and even comorbidity of these two and other autoimmune pathologies [85]. In addition, recent works have also shown that DNA genetic variations largely drive the development and function of specific leukocyte subsets [86], in particular those who may have key pro-inflammatory or regulatory roles in autoimmune diseases [87].

Of note, there is the repeated observation of a sex-related bias in different autoimmune diseases, but often not attributable to known genetic causes [88,89], and that environmental influences at various timepoints contribute to a shift towards unbalanced immune responses [90,91].

Zinc has been recognized as one of these factors, as its homeostasis is essential against inflammatory diseases to regulate different aspects of the immune system, both for innate and adaptive immune response, cell cycle progression, cell maturation and differentiation [92]. Zinc deficiency is therefore associated with an incorrect maturation and function of T and B cells, an unbalanced ratio between Th1 and Th2 [93], and between regulatory and pro-inflammatory T cells, and a weakening of NK cell function. Zinc can inhibit Th17 lymphocytes, which confer susceptibility to autoimmune diseases owing to their strong inflammatory properties, as well as a variety of other proinflammatory responses on T-cells and B-cells [94,95].

These unbalanced states can, however, be restored by zinc integration [96,97,98]. As demonstrated in several studies analyzed in this meta-analysis, patients with multiple sclerosis exhibit low levels of zinc in the plasma [99,100,101]. This is also observed in the mouse model, affected by experimental autoimmune encephalomyelitis (EAE), in which the symptoms decrease in severity and even regress after zinc treatment, inducing proliferation of regulatory T cells and decreasing pro-inflammatory cells [102,103,104,105]. Even in type 1 diabetes, the autoimmune diabetes, zinc homeostasis plays a key role by acting on various molecular mechanisms [106,107]. The protagonist in beta-pancreatic cells is definitely the ZnT8 zinc importer, essential for the transport of insulin secretory vesicles, and for the formation of insulin granules [108,109,110]. Even in this case, the benefits of zinc supplementation are known. Chronic zinc deficiency increases inflammation potentially leading to its chronic perpetuation [5].

Alternatively, hypozincemia could represent a common result of inflammation during the autoimmune disorders here discussed. It has been shown that induction of acute-phase response upregulates Zip14 via IL-6 and IL-1 signaling [111], inducing liver sequestration and redistribution in the cellular compartment [112]. Furthermore, the experiments conducted by Bonaventura and colleagues on synovial cells isolated from joints of patients affected by rheumatoid arthritis are illuminating. The authors have shown that exposure of cells to pro-inflammatory cytokines such as interleukin-17 and tumor necrosis factor alpha, increases the expression of zinc importer carriers, resulting in enhanced intracellular Zn uptake and further increasing inflammation and interleukin-6 production. These experiments have clarified the existence of a feedback loop between inflammation and cellular zinc uptake [113].

Indeed, in pathologies such as multiple sclerosis, serum levels of zinc decrease mainly during relapses; in pathologies characterized by chronic inflammation, such as rheumatoid arthritis, a continuous recruitment of zinc within the cells would be established, hence a continuous depletion of zinc in serum. Probably, in the induction of autoimmunity, there is a role for either a primary zinc deficiency and for its secondary reduction due to inflammation, that warrant further focused studies for a thorough determination of timing (preclinical phase of disease vs. overt disease or during pregnancy, childhood, and elderly), cause/pathophysiology, degree of reduction and span/duration (over time) of hypozincemia. Finally, zinc also acts as a co-factor for many proteins implicated in the epigenome establishment. This means that the development of a new organism may be conditioned, from the earliest stages, by possible imbalance in zinc homeostasis [114]. Therefore, interventions to correct any nutritional imbalances should be anticipated during the stages of pregnancy and lactation. In fact, zinc deficiencies during pregnancy are associated with fetal or adult illness, also due to the improper development of the immune system [115,116,117].

One aspect not to be overlooked is the bioaccessibility and then the bioavailability of this and other elements. In this regard, environmental studies [118,119] are also desirable for helping to clarify the potential environmental impact of exposures/deficiencies to particular elements, essential for/toxic to human health, including any corrective measures to improve the conditions in which man lives and works and therefore having a strong impact on human health. In fact, in the mining exploration field, it is well known that the abundance of some elements is typically linked to some lithologies and/or metallogenic contexts. This relative abundance (or deficiency) affects any media linked to these environments such as water [119,120,121,122,123,124,125,126], soil [89,118,119,124,127,128,129,130,131,132] and so also the biological sphere. However, it should be considered that the complexity and dynamism of the environment could complicate the interpretations [133].

Given the importance of zinc in regulating the functioning of the immune system, it is, therefore, logical to associate an imbalance in the homeostasis of this element with the state of autoimmunity. It would therefore be desirable to mount a screening campaign for the evaluation of zinc levels in neonatal, preschool and school-age children, and hence a relevant campaign for the integration of essential elements for man, including zinc. In populations at higher genetic risk of autoimmunity it would be therefore interesting to have a clinical trial investigate personalized zinc supplementation for preventing and/or treating autoimmune diseases.

## Figures and Tables

**Table 1:** Characteristics of selected studies included in meta-analysis in reference to serum samples.

Authors	Year	Disease	No.		Zn Status (μg/mL)		Direction
			Patients	Controls	Patients	Controls	
Aaseth et al. [20]	1978	RA	22	12	0.654	0.850	low zinc in patients
Abdel Fattah et al. [21]	2016	AA	50	50	0.755	0.857	low zinc in patients
Arreola et al. [22]	1990	T1D	22	11	0.734	1.114	low zinc in patients
Banford et al. [23]	1982	RA	85	49	12.100	12.100	no difference
Bhat et al. [24]	2009	AA	50	50	78.000	88.000	low zinc in patients
Bideci et al. [25]	2005	T1D	28	15	0.961	1.231	low zinc in patients
Brandao-Neto et al. [26]	1999	T1D	10	10	1.040	1.020	no difference
Car et al. [27]	1992	T1D	15	15	0.562	0.772	low zinc in patients
Dijkmans et al. [28]	1987	RA	25	18	0.667	0.942	low zinc in patients
Dore-Duffy et al. [29]	1983	MS	63	62	831.000	817.000	no difference
Dore-Duffyet al. [30]	1990	RA	57	18	0.850	0.997	low zinc in patients
Erdal et al. [31]	2008	HT	43	49	1.093	1.015	no difference
Ghazavi et al. [32]	2012	MS	60	60	0.402	1.278	low zinc in patients
Hagglof et al. [33]	1983	T1D	66	79	0.915	1.000	low zinc in patients
Hansson et al. [34]	1975	RA	37	70	1.066	1.055	low zinc in patients
			42	26	1.052	0.965	low zinc in patients
Haugen et al. [35]	1992	JIA	8	17	0.909	0.981	low zinc in patients
Helgeland et al. [36]	2000	JIA	14	22	0.830	0.870	low zinc in patients
Helliwell et al. [37]	1984	RA	50	50	0.804	0.883	low zinc in patients
Isbir et al. [38]	1994	T1D	20	20	0.565	0.696	low zinc in patients
Jansen et al. [39]	2012	T1D	8	8	0.768	0.883	low zinc in patients
Javanbakht et al. [40]	2012	PV	43	58	0.906	0.988	no difference
Kapaki et al. [41]	1989	MS	15	28	1.030	1.100	no difference
Kiilerich et al. [42]	1986	T1D	7	12	0.798	0.948	low zinc in patients
Kiilerich et al. [43]	1990	T1D	10	104	1.007	0.948	no difference
Kobbah et al. [44]	1988	T1D	30	44	0.785	0.909	low zinc in patients
Lin et al. [45]	2016	T1D	88	76	0.910	0.940	no difference
Iyanda et al. [46]	2011	AA	20	20	0.792	0.933	low zinc in patients
			20	20	0.782	0.933	low zinc in patients
Maldonado et al. [47]	1991	T1D	22	22	1.111	1.197	no difference
Mierzecki et al. [48]	2011	RA	74	30	0.801	0.720	low zinc in patients
Negoro et al. [49]	2004	SS	31	15	0.706	0.866	low zinc in patients
Nicoloff et al. [50]	2005	T1D	35	20	0.675	1.268	low zinc in patients
Onal et al. [51]	2011	RA	32	52	0.430	0.748	low zinc in patients
Palm et al. [52]	1982	MS	21	21	0.850	0.968	low zinc in patients
			29	29	0.791	0.863	low zinc in patients
Raz et al. [53]	1989	T1D	23	22	0.928	0.170	low zinc in patients
Sahebari et al. [54]	2014	SLE	123	100	0.701	0.860	low zinc in patients
Silverio Amancio et al. [55]	2003	JIA	20	10	0.897	0.900	no difference
			21	13	0.976	0.950	no difference
Ullah et al. [56]	2017	RA	61	61	0.856	0.959	low zinc in patients
Yazdanpanah et al. [57]	2011	PV	25	25	0.770	1.207	low zinc in patients
Yilmaz et al. [58]	2005	SLE	27	20	0.875	0.990	low zinc in patients
Zoli et al. [59]	1998	RA	57	20	85.600	108.100	low zinc in patients

Abbreviations: AA, Alopecia Areata; HT, Hashimoto Thyroiditis; JIA, Juvenile Idiopathic Arthritis; MS, Multiple Sclerosis; PV, Pemphigus Vulgaris; RA, Rheumatoid Arthritis; SLE, Systemic Lupus Erythematosus; SS, Sjogren’s Syndrome; T1D, Type 1 Diabetes.

**Table 2:** Characteristics of selected studies included in meta-analysis in reference to plasma samples.

Authors	Year	Disease	No.		Zn Status (μg/mL)		Direction
			Patients	Controls	Patients	Controls	
Arreola et al. [60]	1986	T1D	9	12	73.490	112.460	low zinc in patients
Bacon et al. [61]	1990	JIA	8	9	0.805	0.983	low zinc in patients
		JIA	14	9	0.859	0.983	low zinc in patients
		JIA	12	9	0.875	0.983	low zinc in patients
Balogh et al. [62]	1980	RA	140	100	11.740	15.100	low zinc in patients
Crofton et al. [63]	1983	CD	12	15	0.582	0.974	low zinc in patients
		CD	10	15	0.628	0.974	low zinc in patients
Cunningham et al. [64]	1994	T1D	14	15	0.950	0.910	no difference
Dore-Duffy et al. [29]	1983	MS	68	60	845.000	788.000	low zinc in patients
Dore-Duffy et al. [30]	1990	RA	57	17	0.795	0.890	low zinc in patients
Ho et al. [65]	1986	MS	45	23	0.890	0.880	high zinc in patients
Kennedy et al. [66]	1975	RA	113	100	0.857	0.990	low zinc in patients
Melchior et al. [67]	1989	T1D	14	36	0.947	0.943	no difference
		T1D	12	36	0.879	0.817	no difference
Milanino et al. [68]	1993	RA	120	70	0.895	1.019	low zinc in patients
		RA	10		0.526	0.106	low zinc in patients
Mocchegiani et al. [69]	1989	T1D	15	16	0.793	1.064	low zinc in patients
Naveh et al. [70]	1997	RA	13	8	0.590	1.110	low zinc in patients
		RA	16	8	0.600	1.110	low zinc in patients
Pereira et al. [71]	2011	AH	23	25	0.719	0.807	low zinc in patients
Quilliot et al. [72]	2001	T1D	25	20	0.940	0.970	low zinc in patients
Rohn et al. [73]	1993	T1D	45	12	0.942	0.981	no difference
Ruiz et al. [74]	1998	T1D	16	9	1.020	1.079	no difference
		T1D	13	7	1.046	1.059	no difference
		T1D	31	19	1.020	1.040	no difference
		T1D	34	24	1.046	1.040	no difference
		T1D	31	17	1.033	1.046	no difference
		T1D	25	14	1.013	1.059	no difference
Smith et al. [75]	1989	MS	27	33	0.987	1.000	no difference
Tuncer et al. [76]	1999	RA	38	20	1.087	1.253	low zinc in patients
Viktorinova et al. [77]	2009	T1D	11	34	0.885	0.942	no difference
Yazar et al. [78]	2005	RA	25	25	0.663	0.658	no difference

Abbreviations: AH, Autoimmune Hepatitis; Cd, Celiac Disease; JIA, Juvenile Idiopathic Arthritis; MS, Multiple Sclerosis; RA, Rheumatoid Arthritis; T1D, Type 1 Diabetes.

**Table 3:** Characteristics of selected studies included in meta-analysis in reference to hair, urine and CSF samples.

Authors	Year	Disease	No.		Biological Sample	Zn Status (μg/g)		Direction
			Patients	Controls		Patients	Controls	
Afridi et al. [79]	2015	RA	15	14	Hair	122.00	178.00	low zinc in patients
			15	12		117.00	167.00	low zinc in patients
			12	13		135.00	203.00	low zinc in patients
			11	13		126.00	203.00	low zinc in patients
Afridi et al. [80]	2012	RA	39	47	Hair	112.00	225.00	low zinc in patients
			34	52		138.00	250.00	low zinc in patients
			23	22		122.00	178.00	low zinc in patients
			20	19		135.00	203.00	low zinc in patients
Hagglof et al. [33]	1983	T1D	74	30	Hair	160.90	190.80	low zinc in patients
Mierzecki et al. [48]	2011	RA	71	75	Hair	150.37	150.37	no difference
Kiilerich et al. [43]	1990	T1D	10	28	Urine	1006.85	509.96	high zinc in patients
Milanino et al. [68]	1993	RA	75	50	Urine	437.9	457.50	no difference
Maldonado et al. [47]	1991	T1D	13	8	Urine	353	984.00	low zinc in patients
Naveh et al. [70]	1997	RA	16	8	Urine	538	984.00	low zinc in patients
			22	22	Urine	1396	611.00	high zinc in patients
Kapaki et al. [41]	1989	MS	15	28	CSF	34.73	34.70	no difference
Melo et al. [81]	2003	MS	18	19	CSF	19.00	23.50	no difference

Abbreviations: MS, Multiple Sclerosis; RA, Rheumatoid Arthritis, T1D, Type 1 Diabetes.

## Supplementary Materials

The following are available online at http://www.mdpi.com/2072-6643/10/1/68/s1. Table S1, Full search details for all databases; Table S2, List of the 13 papers excluded because not possible to retrieve with the means available to the Italian library system; Figure S1, (a) Temporal distribution of the 62 publications included in the meta-analysis; (b) Temporal distribution of publications related to the most frequent pathologies.
