# Supplementary material for: Zinc Status and Autoimmunity: A Systematic Review and Meta-Analysis
Source: Nutrients. 2018 Jan 11;10(1):68. doi: 10.3390/nu10010068 (PMC5793296; doi:10.3390/nu10010068)
Supplement: Supplementary file 1 [file nutrients-10-00068-s001.pdf]

Table S1. Full search details for all databases

Database	Keywords	Papers founded
PubMed	Zinc + dietary + Autoimmune Diseases	106
	Zinc + supplement + Autoimmune Diseases	34
	Zinc + serum + Autoimmune Diseases	167
	Zinc + plasma + Autoimmune Diseases	126
	Zinc + dietary + Autoimmunity	21
	Zinc + supplement + Autoimmunity	2
	Zinc + serum + Autoimmunity	16
	Zinc + plasma + Autoimmunity	10
Cochrane Central Register of Controlled Trials	Zinc + dietary + Autoimmune Diseases	1
	Zinc + supplement + Autoimmune Diseases	1
	Zinc + serum + Autoimmune Diseases	0
	Zinc + plasma + Autoimmune Diseases	0
	Zinc + dietary + Autoimmunity	2
	Zinc + supplement + Autoimmunity	1
	Zinc + serum + Autoimmunity	1
	Zinc + plasma + Autoimmunity	0
Web of Science	Zinc + dietary + Autoimmune Diseases	12
	Zinc + supplement + Autoimmune Diseases	3
	Zinc + serum + Autoimmune Diseases	67
	Zinc + plasma + Autoimmune Diseases	23
	Zinc + dietary + Autoimmunity	11
	Zinc + supplement + Autoimmunity	4
	Zinc + serum + Autoimmunity	45
	Zinc + plasma + Autoimmunity	12
Science Direct	Zinc + dietary + Autoimmune Diseases	3,167
	Zinc + supplement + Autoimmune Diseases	3,354
	Zinc + serum + Autoimmune Diseases	6,980
	Zinc + plasma + Autoimmune Diseases	5,790
	Zinc + dietary + Autoimmunity	946
	Zinc + supplement + Autoimmunity	976
	Zinc + serum + Autoimmunity	2,314
	Zinc + plasma + Autoimmunity	1,903

Table S2. List of the 13 papers excluded because not possible to retrieve with the means available to the Italian library system

Title	Authors	Year	Source
Penicillamine in rheumatoid arthritis. Connective tissue changes and alterations in serum copper and phase reactants in relation to clinical improvement.	Hansen, T.M.; Manthorpe, R.; Kofod, B.; Andreassen, T.; Oxlund, H.; Lorenzen, I.B.	1976	J Rheumatol. 3, 367-74.
Plasma zinc levels in multiple sclerosis.	Wong Jr.; E.K.; Enomoto, H.; Leopold, I.H.; Fleischer, E.B.; Schoon, D.V.; Fender, D.; Tucker, H.G.; Adamson, B.; Kladde, L.; Kazan, D.; Nudleman, K.	1980	Metabolic and pediatric ophthalmology, 4, 3-8.
Serum copper and zinc in rheumatoid arthritis and osteoarthritis.	Grennan, D.M.; Knudson, J.M.L.; Dunckley, J.; MacKinnon, M.J.; Myers, D.B.; Palmer, D.G.	1980	New Zealand Medical Journal, 91, 47-50.
Zinc nutriture in type I diabetes mellitus: relationship to growth measures and metabolic control.	Canfield, W.K.; Hambidge, K.M.; Johnson, L.K.	1984	J Pediatr Gastroenterol Nutr. 3, 577-584
Magnesium and zinc in diabetic pregnancy.	Wibell, L.; Gebre-Medhin, M.; Lindmark, G.	1985	Acta Paediatrica Scandinavica, 74, 100-106.
Trace elements (copper, zinc and iron) in serum of rheumatic children living in south-western Finland.	Hyörä, H.; Mäkelä, A.; Pakarinen, P.; Bergman, T.; Näntö, V.	1986	Acta Pharmacologica et Toxicologica, 59, 403-405.
Effects of chronic and acute corticosteroid therapy on zinc and copper status in rheumatoid arthritis patients.	Peretz, A.; Neve, J.; Famaey, J.P.	1989	Journal of trace elements and electrolytes in health and disease, 3, 103-108.
Vitamins A and E, retinol binding protein and zinc in rheumatoid arthritis.	Honkanen, V.; Konttinen, Y.T.; Mussalo-Rauhamaa, H.	1989	Clinical and experimental rheumatology, 7, 465-469.
Serum zinc, copper and selenium in rheumatoid arthritis.	Honkanen, V.; Konttinen, Y.; Sorsa, T.; Hukkanen, M.; Kemppinen, P.; Santavirta, S.; Saari, H.; Westermarck, T.	1991	Journal of trace elements and electrolytes in health and disease, 5, 261-263.
Effect of a strict vegan diet on energy and nutrient intakes by Finnish rheumatoid patients.	Rauma, A.; Nenonen, M.; Helve, T.; Hanninen, O.	1993	European Journal of Clinical Nutrition, 47, 747-749.
Low plasma zinc levels in active rheumatoid arthritis.	Weinstein, A	1998	The Journal of rheumatology, 25, 187-188.
Copper, zinc and magnesium levels in type-1 diabetes mellitus.	Zargar, A.H.; Bashir, M.I.; Masoodi, S.R.; Laway, B.A.; Wani, A.I.; Khan, A.R.; Dar, F.A.	2002	Saudi Medical Journal, 23, 539-542.
New potential serum biomarkers in multiple sclerosis identified by proteomic strategies.	Amin, B.; Maurer, A.; Voelter, W.; Melms, A.; Kalbacher, H.	2014	Current medicinal chemistry, 21, 1544-1556.

Figure S1

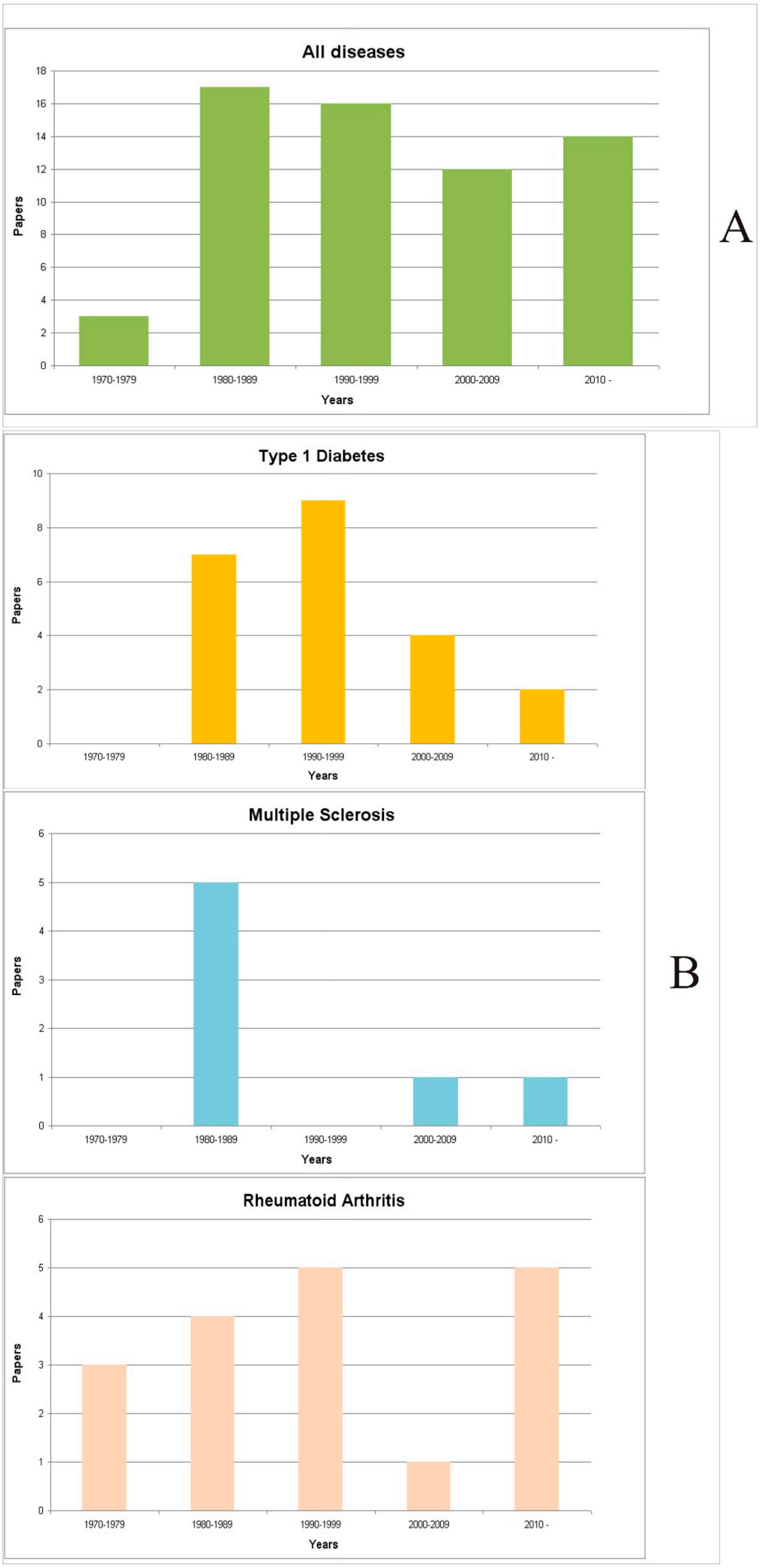
All diseases
Papers
Years
1970-1979
1980-1989
1990-1999
2000-2009
2010 -
A
Type 1 Diabetes
Multiple Sclerosis
Rheumatoid Arthritis
B
